# Diagnostic performance of ^68^Gallium-PSMA-11 PET/CT to detect significant prostate cancer and comparison with ^18^FEC PET/CT

**DOI:** 10.18632/oncotarget.22441

**Published:** 2017-11-14

**Authors:** Manuela A. Hoffmann, Matthias Miederer, Helmut J. Wieler, Christian Ruf, Frank M. Jakobs, Mathias Schreckenberger

**Affiliations:** ^1^ Supervisory Center for Medical Radiation Protection, Bundeswehr Medical Service Headquarters, Koblenz, Germany; ^2^ Department of Nuclear Medicine, Johannes Gutenberg-University, Mainz, Germany; ^3^ Department of Nuclear Medicine, Bundeswehr Central Hospital, Koblenz, Germany; ^4^ Department of Urology, Bundeswehr Central Hospital, Koblenz, Germany; ^5^ Department of Epidemiology, German Air Force Center for Aerospace Medicine, Fürstenfeldbruck, Germany

**Keywords:** prostate cancer, positron emission tomography/computed tomography, prostate-specific membrane antigen, choline, detection of significant cancer

## Abstract

**Background:**

Radiolabeled prostate-specific membrane antigen (PSMA) has proven to be a highly accurate method to detect recurrence and metastases of prostate cancer, but only sparse data is available about its performance in the diagnosis of clinically significant primary prostate cancer.

**Methods:**

We compared ^68^Ga-PSMA-11 PET/CT in 25 patients with ^18^FEC PET/CT in 40 patients with suspected prostate carcinoma based on an increased PSA level.

The PET/CT results were compared with the histopathologic Gleason Score (GS) of biopsies.

**Results:**

The ^68^Ga-PSMA-11 PET/CT revealed highly suspect prostatic lesions (maximum standardized uptake value/SUV_max_ >2.5) in 21/25 patients (84%), associated with GS≥6 (low-grade/high-grade carcinoma). Two histopathologic non-malignancy-relevant cases (GS<6) had PSMA-SUV_max_ ≤2.5; all histopathologic high-grade cases (GS≥7b) showed PSMA-SUV_max_ >12.0 which further increased with rising GS. There were 2 false positives and no false negative findings for high-grade prostate cancer using a cut off-level for SUV_max_ of 2.5.

In contrast, the ^18^FEC PET/CT showed suspected malignant lesions in 38/40 patients (95%), which included 3 lesions with GS<6. The mean SUV_max_ values did not differ with different GS. There were 11 false positives and 1 false negative for detection of high-grade prostate cancer (cut off 2.5).

By means of ROC analysis a SUV_max_ of 5.4 was found to be an optimal cut off-level to distinguish between low- and high-grade carcinoma in ^68^Ga-PSMA-11 PET/CT (AUC=0.9692; 95% CI 0.9086;1.0000;SD(AUC)=0.0309)). Choosing a cut off-level of SUV_max_5.4, ^68^Ga-PSMA-11 PET/CT was able to distinguish between GS ≤7a/≥7b with a sensitivity of 84%, a specificity of 100%, a negative predictive value (NPV) of 67%, and an efficiency of 88% (*p*<0.001).

The ROC analysis revealed a SUV_max_ 6.5 as an optimal cut off-level to distinguish between low- and high-grade carcinoma in ^18^FEC PET/CT (AUC=0.7470; 95% CI 0.5919;0.9020;SD(AUC)=0.0791) with a sensitivity of 61% and a specificity of 92%; but the efficiency was only 70% and the NPV 50% (*p*=0.01).

**Conclusion:**

^68^Ga-PSMA-11 PET/CT guided biopsy of the prostate increases diagnostic precision and is likely to help to reduce overtreatment of low-grade malignant disease as well as detect the foci of the highest Gleason pattern. Both methods (^68^Ga-PSMA-11,^18^FEC) were suitable to detect primary prostate cancer, but the excellent image quality, the higher specificity and the good correlation of positive scans with GS are advantages of ^68^Ga-PSMA-11.

## INTRODUCTION

Exact diagnosis and staging of primary prostate cancer is crucial for optimal treatment decisions. The differentiation between an indolent low-grade tumor (GS 6, 7a) which may allow conservative management such as active surveillance and an aggressive high-grade tumor (GS≥7b) [1–3] leading to prostatectomy, radio-/chemotherapy or androgen-deprivation is essential for the patient’s quality of life and prognosis. Epstein and Montironi showed that GS 7 is misleading, as GS7b tumors have a much worse prognosis than GS7a tumors and should be considered differently for treatment and prognostic purposes [2].

Morphological imaging techniques, e.g. transrectal ultrasound, computed tomography and magnetic resonance imaging, have limited accuracy in the diagnosis of primary prostate cancer [4, 5]. Imaging is the preferred base for sampling biopsies to minimize undetected lesions leading to under treatment [6–9], and over diagnosis resulting in overtreatment of low risk lesions [10].

Molecular imaging with specific tracers should improve diagnostic accuracy. Some studies report a high sensitivity of PET/CT with radioactive labeled choline derivatives for the detection of primary prostate cancer lesions by focally increased choline uptake [11–14]. But although choline PET/CT is widely used, other studies report low sensitivity and specificity, particularly at low PSA levels and high GS [15–19]. The major factors which compromise the diagnostic accuracy of choline PET/CT for primary prostate cancer are the tumor configuration, especially the detection of small carcinoma, and the differentiation of prostatitis, benign prostatic hyperplasia or high-grade intraepithelial neoplasia [4, 5, 13, 20, 21].

Prostate-specific membrane antigen (PSMA) is a transmembrane protein which is significantly overexpressed in prostate carcinoma cells, and its expression increases with tumor aggressiveness [4, 5, 13, 20, 21], metastatic disease and recurrence while normal prostatic tissue expresses PSMA sparsely [22–24]. PSMA PET/CT and multiparametric magnetic resonance imaging (mpMRI) correlated well with regard to tumor allocation in patients with a high pretest probability for large tumors [25]. Preliminary results of 37 patients with recurrent prostate cancer and rising PSA levels (background ratio between 18.8 and 28.3) indicated that the labeling of PSMA ligand with ^68^Ga could detect relapses and metastases of prostate carcinoma with high contrast compared to normal tissue [23].

Afshar-Oromieh et al. (2014) used an ^68^Ga-labeled HBED-CC conjugate of the PSMA-specific pharmacophore Glu-NH-CO-NH-Lys (“^68^Ga-PSMA-11”), and detected at least one lesion characteristic for prostate cancer in 86.5% of patients, but only in 26 of 37 (70.3%) patients with ^18^FEC PET/CT. In patients with PSA values ≤2.82 ng/ml at least one lesion characteristic of prostate cancer was identified in 68.8% of patients with ^68^Ga-PSMA-11 PET/CT, but only in 43.8% of patients with ^18^FEC PET/CT. All lesions detected by ^18^FEC PET/CT were also seen in ^68^Ga-PSMA-11 PET/CT [19].

Despite positive results for recurrent prostate cancer the availability of data for the primary diagnosis is poor. Therefore, the aim of this study was the evaluation of the diagnostic performance of^68^Ga-PSMA-11 PET/CT for detection of primary prostate cancer in patients with increased PSA levels and comparison of the results to ^18^FEC PET/CT. The intent of this study is to improve the diagnostic precision of prostate cancer detection in patients with increased PSA by use of ^68^Ga-PSMA-11 PET/CT with subsequent imaging guided biopsy. Furthermore, we have demonstrated that this approach may be able to predict the histological aggressiveness of the underlying tumor.

## RESULTS

^68^Ga-PSMA-11 PET/CT was conducted in 25 patients with a mean age of 67.0 ± 8.1 years and a basic PSA of 20.4 ± 33.50 ng/ml. Another 40 patients, who underwent ^18^FEC PET/CT, were aged 69.4 ± 7.7 years and had a mean basic PSA of 55.0 ± 56.9 ng/ml (Table 1).

**Table 1:** Characteristics of patients with suspected prostate cancer

Test	^68^Ga-PSMA-11 PET/CT		^18^FEC PET/CT	
	n	(mean ± SD)	n	(mean ± SD)
Age (years)	25	67.0 ± 8.1	40	69.4 ± 7.7
PSA (ng/ml)	25	20.4 ± 33.5	40	55.0 ± 56.9
SUV_max_ prostate	25	12.1 ± 13.9	40	6.3 ± 6.4
Patients with metastases	7		15	
Patients without metastases	18		25	
SUV_max_ positive lymph nodes	4	16.9 ± 12.5	13	11.0 ± 11.5
SUV_max_ bone metastases	4	24.8 ± 25.9	8	7.7 ± 7.7
SUV_max_ lung metastases	1	2.9	1	-
Perineural invasion	8		10	

SD, standard deviation.

**Figure 1:**
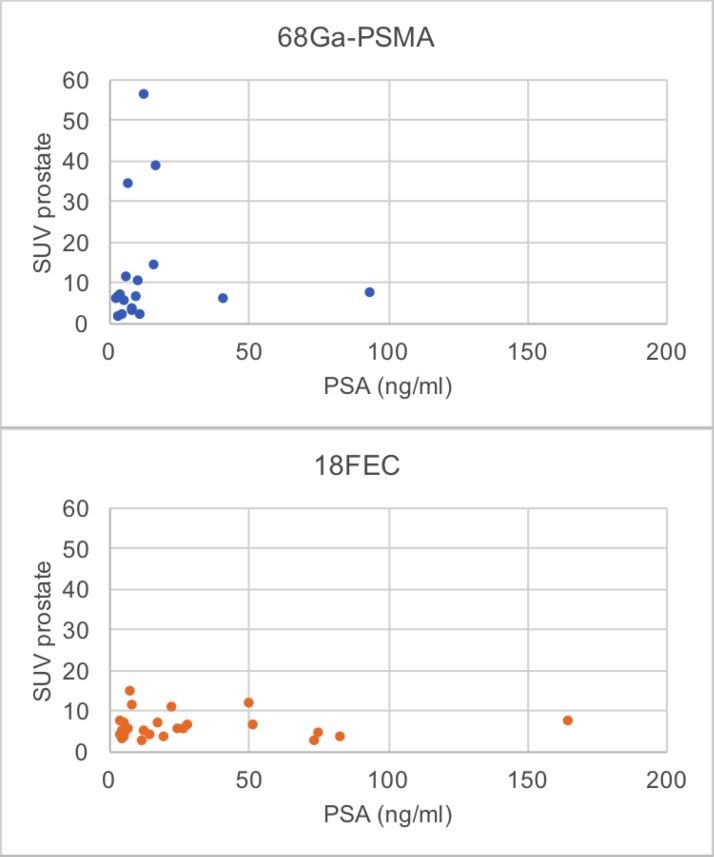
Relation between PSA and prostatic SUV_max_ of ^68^Ga-PSMA-11 PET/CT and ^18^FEC PET/CT in patients without metastases (^68^Ga-PSMA PET/CT: R 0.42, p= 0.082; ^18^FEC PET/CT: R 0.033; p = 0.875)

The ^68^Ga-PSMA PET/CT scans showed prostatic lesions with accumulation of the radiotracer above SUV_max_ 2.5 in 21/25 patients (84%), which related to lesions with GS≥6 (low-grade and high-grade carcinoma). The mean prostatic SUV_max_ was 12.1 ± 13.9 (1.5-56.0) in the whole group and 12.1 ± 14.7 (1.5-38.7) in 18 patients without metastases. In the group of ^18^FEC PET/CT scans such lesions (SUV_max_ >2.5, GS≥6) were seen in 38/40 patients (95%) and the mean prostatic SUV_max_ accounted for 6.3 ± 6.4 in the whole group and 5.8 ± 2.8 (2.4-14.5) in 25 patients without metastases. In ^68^Ga-PSMA PET/CT, but not in ^18^FEC PET/CT, there was a tendency towards increasing SUV_max_ with rising PSA as shown in Figure 1 for patients without metastases (^68^Ga-PSMA PET/CT: R 0.42, p = 0.082; ^18^FEC PET/CT: R 0.033; p = 0.875).

After histopathologic examination of biopsies and application of the GS, 8% of patients in the ^68^Ga-PSMA PET/CT group had non-malignancy-relevant (GS<6), 16% low-grade (GS 6, 7a) and 76% high-grade (GS≥7b) lesions. The corresponding distribution in the ^18^FEC PET/CT-group was 10%, 20%, and 70%, respectively (Table 2). Rising SUV_max_ values were associated with rising GS categories in both ^68^Ga-PSMA PET/CT (R 0.642, p = 0.005) and ^18^FEC PET/CT (R 0.404, p = 0.009).

**Table 2:** Distribution of GS in two groups of patients with ^68^Ga-PSMA PET/CT or ^18^FEC PET/CT using different SUV_max_ cut off-levels

^68^Ga-PSMA (n = 25)	Gleason Score					
	<6	6	7a	7b	8	9
	Non- malignancy- relevant	Low-grade carcinoma		High-grade carcinoma		
**SUV ≤2.5**	2 50.0%	1 25.0%	1 25.0%	0	0	0
**SUV >2.5**	0	1 4.8%	1 4.8%	9 42.9%	4 19.1%	6 28.6%
Total	2 8.0%	2 8.0%	2 8.0%	9 36.0%	4 16.0%	6 24.0%
**SUV ≤5.4**	2 22.2%	2 22.2%	2 22.2%	2 22.2%	1 11.1%	0
**SUV >5.4**	0	0	0	7 43.8%	3 18.8%	6 37.5%
Total	2 8.0%	2 8.0%	2 8.0%	9 36.0%	4 16.0%	6 24.0%

^18^FEC (n = 40)	Gleason Score					
	<6	6	7a	7b	8	9
	Non- malignancy- relevant	Low-grade carcinoma		High-grade carcinoma		
**SUV ≤2.5**	1 50.0%	0	0	0	1 50.0%	0
**SUV >2.5**	3 7.9%	3 7.9%	5 13.2%	7 18.4%	6 15.8%	14 36.8%
Total	4 10.0%	3 7.5%	5 12.5%	7 17.5%	7 17.5%	14 35.0%
**SUV ≤6.5**	4 18.2%	2 9.1%	5 22.7%	2 9.1%	4 18.2%	5 22.7%
**SUV >6.5**	0	1 5.6%	0	5 27.8%	3 16.7%	9 50.0%
Total	4 10.0%	3 7.5%	5 12.5%	7 17.5%	7 17.5%	14 35.0%

**Figure 2:**
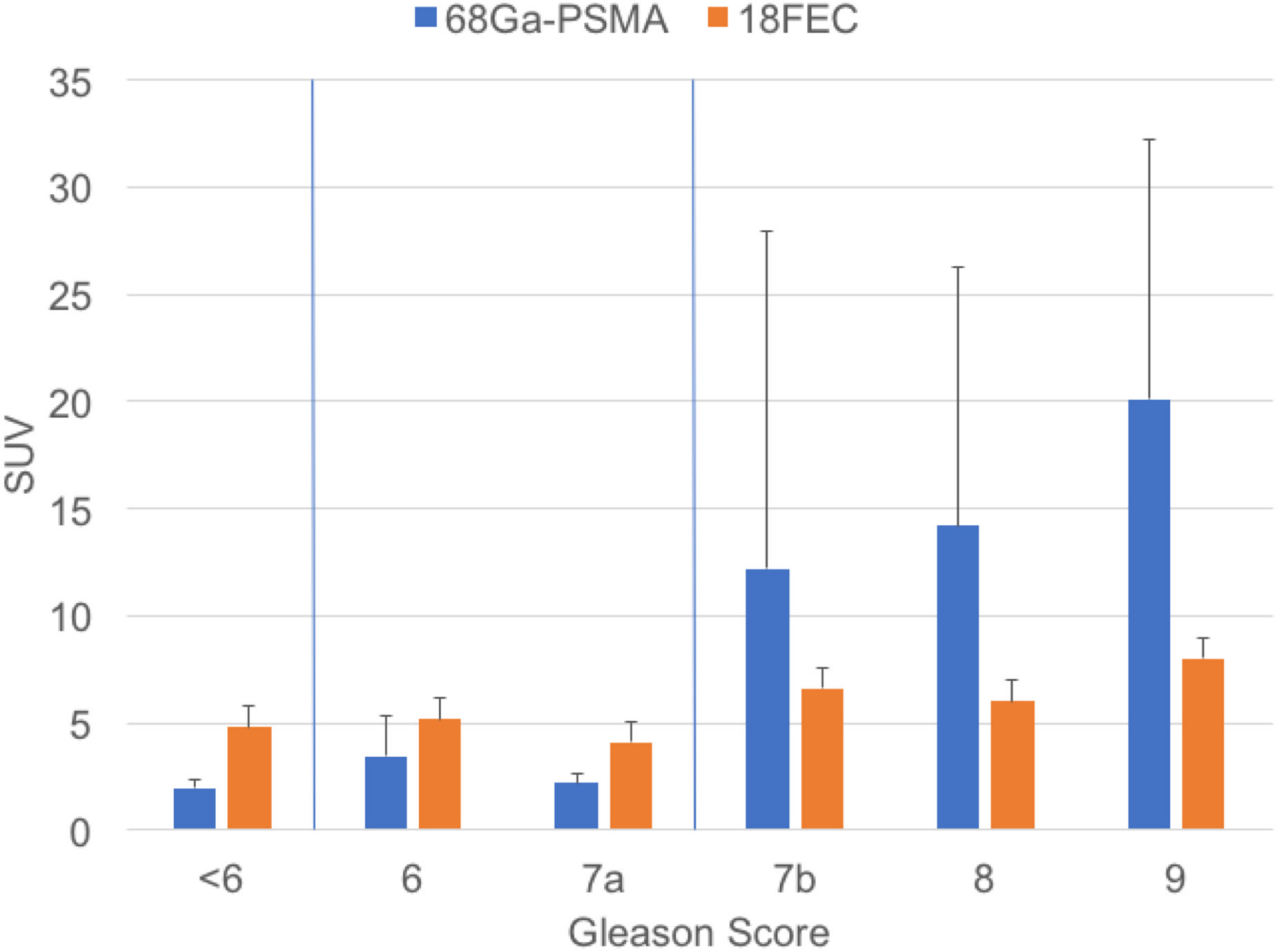
Distribution between GS and SUV_max_in ^68^Ga-PSMA PET/CT scans and ^18^FEC PET/CT scans for prostate cancer

As shown in Figure 2 the two histopathologic non-malignancy-relevant lesions (GS<6) in the ^68^Ga-PSMA PET/CT group had SUV_max_≤2.5 and the SUV_max_ means of all histopathologic high-grade categories (GS≥7b) continuously increased starting from SUV_max_ values above 12.0, whereas in the ^18^FEC PET/CT group the SUV_max_ means remained stable across all GS categories.

Using an SUV_max_ of 2.5 as the cut off-level between not-malignancy-relevant and malignant lesions (GS<6 vs. GS≥6) ^68^Ga-PSMA PET/CT revealed no false malignant and 2/23 false non-malignancy-relevant (8.7%) results indicating a sensitivity of 91%, a specificity of 100% and a NPV of 50%. With regard to the distinction between low-grade (GS≤7a) and high-grade carcinoma (GS≥7b) the sensitivity was 100%, the specificity 67% and the NPV 100%.

**Figure 3:**
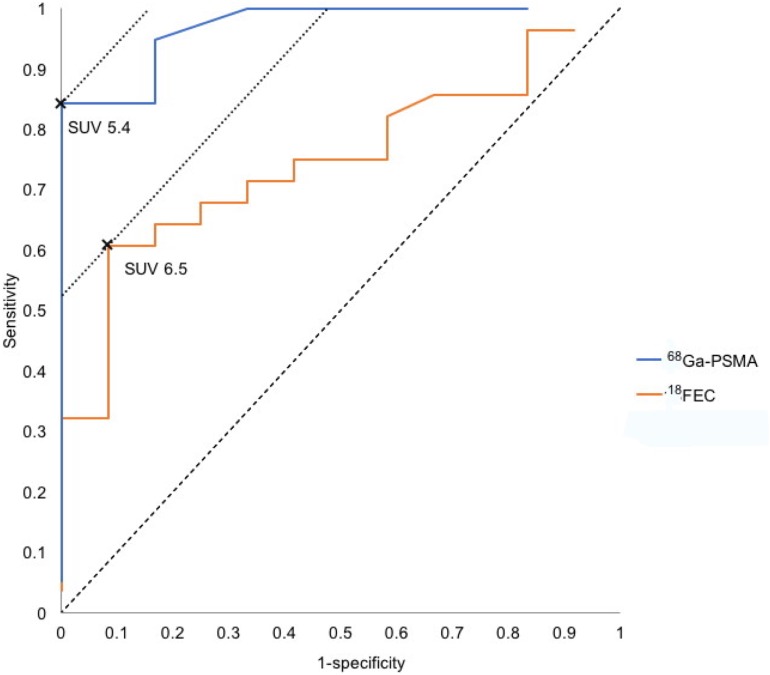
ROC curve according to the SUV_max_ values of our patients

Using ROC analysis SUV_max_ 5.4 was found to be an optimal cut off-level to distinguish between low- and high-grade carcinoma by means of ^68^Ga-PSMA PET/CT (AUC = 0.9692; 95% CI 0.9086; 1.0000; SD(AUC) = 0.0309) (Figure 3). Applying this split-point analysis the specificity increased to 100% while the sensitivity was 84%, which resulted in an efficiency (= sum of all correct diagnoses) of 88% and a NPV of 67% (*p*<0.001) (Table 3).

**Table 3:** Test parameters for ^68^Ga-PSMA PET/CT and ^18^FEC PET/CT

	*GS<6 vs. ≥6* *Cut off SUV*_*max*_ *2.5*		*GS≤7a vs. ≥7b* *Cut off SUV*_*max*_ *2.5*		*GS≤7a vs. ≥7b* *Optimal SUV*_*max*_ Cut off^*^	
	^*68*^*Ga-PSMA*	^*18*^*FEC*	^*68*^*Ga-PSMA*	^*18*^*FEC*	^*68*^*Ga-PSMA*	^*18*^*FEC*
*Sensitivity*	*91%*	*97%*	*100%*	*96%*	*84%*	*61%*
*Specificity*	*100%*	*25%*	*67%*	*8%*	*100%*	*92%*
*NPV*	*50%*	*50%*	*100%*	*50%*	*67%*	*50%*
*PPV*	*100%*	*92%*	*90%*	*71%*	*100%*	*94%*
*Efficiency*	*92%*	*90%*	*92%*	*70%*	*88%*	*70%*

^*68^Ga-PSMA PET/CT SUV_max_ 5.4, ^18^FEC PET/CT SUV_max_ 6.5.

In comparison, at a chosen split-point of SUV_max_ 2.5 the sensitivity and specificity of ^18^FEC PET/CT was 97% and 25% for all malignant lesions (GS≥6) and 96% and 8% for the separation between GS≤7a vs. ≥7b. The ROC analysis revealed a SUV_max_ 6.5 as an optimal cut off-level (AUC = 0.7470; 95% CI 0.5919; 0.9020; SD(AUC) = 0.07910) (Figure 3) with a sensitivity of 61% and a specificity of 92%; but the efficiency was only 70% and the NPV 50% (*p*=0.01) (Table 3).

In 7 patients ^68^Ga-PSMA PET/CT showed metastases in lymph nodes (n = 4), bones (n = 4) and/or lung (n = 1). Also biopsy results demonstrated perineural invasion in 8 of 25 patients, which was associated with PET-positive nodal metastases in 2 patients, but with no metastases in 6 patients.

The ^18^FEC scans showed metastases in 15 patients, mainly in the lymph nodes (n = 13) and bones (n = 8). A lung metastasis was found in one patient. In 10 patients the biopsy results revealed perineural invasion, which was associated with nodal metastases in five cases. Another 5 patients had a perineural invasion without metastases.

## DISCUSSION

In this study, we present the results from two groups of patients with increased PSA plasma levels undergoing ^68^Ga-PSMA-11 PET/CT (n = 25) or ^18^FEC PET/CT (n = 40) and imaging guided biopsy to detect significant cancer of the prostate.

^18^FEC PET/CT has been in widespread use for the diagnosis of prostate carcinoma [19], but in many malignant lesions the choline metabolism is not increased while most prostate carcinomas overexpress PSMA [19]. By labeling of PSMA ligands with ^68^Ga, relapses and metastases of prostate carcinoma with high contrast compared to normal tissues can be detected [23]. Therefore, ^68^Ga-PSMA PET/CT might also be superior to ^18^FEC PET/CT in the detection of primary prostate cancer.

In two groups of patients with elevated PSA levels, scheduled for biopsy, we analyzed ^68^Ga-PSMA-11 PET/CT-scans of 25 patients with an increased PSA and compared the results to ^18^FEC PET/CT scans of another 40 patients. Prostatic lesions with radiotracer uptake above SUV_max_ 2.5 were seen in 21/25 patients (84%) with ^68^Ga-PSMA-11 PET/CT scans and in 38/40 patients (95%) with ^18^FEC PET/CT. In the patient group with and without metastases both ^68^Ga-PSMA-11 PET/CT scans and ^18^FEC PET/CT scans showed no tendency towards increasing SUV_max_ with rising PSA. However in the patient group without metastases ^68^Ga-PSMA PET/CT, but not ^18^FEC PET/CT, showed a tendency towards increasing SUV_max_ with rising PSA (^68^Ga-PSMA PET/CT: R 0.42, p = 0.082; ^18^FEC PET/CT: R 0.033; p = 0.875).

Choosing an SUV_max_ of 2.5 as cut off-level between histologically confirmed non-malignancy-relevant and malignant lesions (GS<6/≥6), ^68^Ga-PSMA-11 PET/CT detected 2/2 cases (100%) as correct benign, but ^18^FEC PET/CT only 1/4 (25%). False negative ^68^Ga-PSMA-11 PET/CT scans (not used in our study) may occur if a prostate tumor is poorly differentiated and displays neuroendocrine aberrations [23, 26]. The specificity of 100% for ^68^Ga-PSMA-11 PET/CT and of 25% for ^18^FEC PET/CT for differentiating between non-malignancy-relevant and malignancy-relevant lesions (GS<6/≥6) observed here, has to be interpreted with caution because of the low rate of benign results in both groups. Our study was conducted in patients with a high probability of malignancy due to an increased PSA which is regarded as a marker for progressive disease because there is a strong association between PSA-level and positive^68^Ga-PSMA-11 PET/CT scans [27] but not with ^18^FEC PET/CT [12]. Studies including a higher proportion of patients with benign prostate alterations are needed to verify the results.

In contrast, the calculation of sensitivity and other prognostic parameters did not show major differences between both radio tracer methods using a cut off-level of 2.5. ^68^Ga-PSMA-11 PET/CT detected 21/23 cases (91%) and ^18^FEC PET/CT 35/36 (97%) correctly identified malignancy (GS≥6) (Table 3). However, for discrimination between high-grade and low-grade carcinoma (GS≤7a/GS≥7b), ^68^Ga-PSMA-11 PET/CT was superior to ^18^FEC PET/CT.

In our study we distinguished between non-malignancy-relevant (GS<6) and malignant (GS≥6) and between low-grade carcinoma (GS≤7a) and high-grade carcinoma (GS≥7b). The latter for the reason, that several studies showed significantly worse prognosis with regard to prostate cancer with GS≥7b [27].

With regard to prognosis and the choice of treatment individual risk stratification is of great importance. Afshar-Oromieh et al. (2015) found no correlation between ^68^Ga-PSMA PET/CT-results and GS [27] in spite of such correlation having been described in the literature [28–31]. They explained this by their low number of patients with a GS 5–6 which might have caused substantial variability in the statistical analysis. In our study we observed a possible correlation between GS and the SUV_max_ values measured by ^68^Ga-PSMA PET/CT: The two histopathologic non-malignancy-relevant lesions (GS<6) had PSMA-SUV_max_ ≤2.5 and all histopathologic high-grade lesions (GS≥7b) showed PSMA-SUV_max_ >2.5. Starting from GS 7b the mean SUV_max_ increased with rising GS category, while the SUV_max_ means of ^18^FEC PET/CT scans did not vary between different GS categories. Statistical analysis revealed a correlation between SUV_max_ and GS for both methods (^68^Ga-PSMA PET/CT R 0.642, p = 0.005; ^18^FEC PET/CT R 0.404, p = 0.009). However the sensitivity for distinction between GS≤7a/≥7b by means of ^68^Ga-PSMA-11 PET/CT vs. ^18^FEC PET/CT at a cut off-level of SUV_max_ 2.5 was 100% vs. 96%, the specificity 67% vs. 8%, NPV 100% vs. 50% and PPV 90% vs. 71%. This means that only ^68^Ga-PSMA-11 PET/CT was able to correctly predict an aggressive prostate cancer.

Assuming that a greater amount of prostate tissue is altered in higher GS categories this difference might be due to a higher specificity of the radiolabeled ^68^Ga-PSMA for cancer cells than ^18^FEC. Because of the high affinity of PSMA to prostate cancer cells every lesion with an accumulation of PSMA should be regarded as prostate cancer or prostate cancer metastasis until proven otherwise [27]. This means that a significant focal increase of PSMA metabolism might be predictive for a high GS, i.e. an aggressive cancer.

Our data showed that ROC analysis indicated an optimal cut off-level of SUV_max_ 5.4 for ^68^Ga-PSMA-11 PET/CT, which increased the specificity and PPV for separating GS≤7a/≥7b up to 100% but slightly decreased sensitivity to 84% and NPV to 67%. Using the calculated optimal cut off-level of SUV_max_ 6.5 for ^18^FEC PET/CT all prognostic parameters were less favorable than for ^68^Ga-PSMA-11 PET/CT.

Fendler et al. (2016) evaluated the accuracy of ^68^Ga-PSMA-11 PET/CT to localize cancer in the prostate and surrounding tissue. They found a statistically higher SUV_max_ in histopathologically positive segments (11.8 ± 7.6) compared to negative segments (4.9 ± 2.9; *p*<0.001). ROC revealed an optimal SUV_max_ cut off-value of 6.5 for discrimination between positive/negative segments [32], i.e. the results were concordant with our data. Fendler et al. concluded that ^68^Ga-PSMA-11 PET/CT accurately detects location and extent of primary prostate cancer and might be a promising tool for noninvasive tumor characterization and biopsy guidance [32]. Koerber et al. observed a significantly higher mean SUV_max_ in tumors with higher d`Amico risk classification and GS from biopsy (*p*<0.001 for grouped analyses) [33]. Giesel et al. published that based on the eight-segment resolution of biopsy, mpMRI and PSMA PET/CT presented identical tumor allocations [25]. MpMRI combined with ultrasound fusion guided biopsy of the prostate is a widely accepted method for T-Staging. However, no statistical significance could be shown regarding the differentiation between high- and low-grade carcinomas in a recently published study by our group [34].

High PSA values, T2b-T3 stage, poor tumor differentiation and perineural invasion are associated with high risk of nodal metastases [35, 36]. Measurement of PSA alone is not helpful in predicting lymph node metastases [37]. With regard to the therapeutic strategy, T-staging determines local surgery and radiotherapy while the pretreatment nodal status defines the extent of pelvic lymph node dissection or radiotherapy [38, 39]. PSMA PET/CT may provide valuable information in planning for focal radiation to the dominant lesions [40], because of its ability to detect even small lymph metastases, primarily due to a high radiotracer uptake [19].

In our study ^68^Ga-PSMA-11 PET/CT revealed metastases in 7/25 patients (28%) and ^18^FEC PET/CT in 15/40 patients (37.5%), mainly in lymph nodes and bone. In 4 patients positive lymph nodes, in 4 patients bone metastases and in 1 patient a lung metastasis were demonstrated by ^68^Ga-PSMA-11 PET/CT. Whereas by ^18^FEC PET/CT, 13 patients showed positive lymph nodes, 8 patients bone metastases and 1 patient a lung metastasis. A perineural invasion was seen in 8 patients (biopsy results according to ^68^Ga-PSMA-11 PET/CT) and in 10 patients (biopsy results according to ^18^FEC PET/CT), respectively. But a perineural invasion was associated with nodal metastases in only 2 of 8 (^68^Ga-PSMA-11 PET/CT) and 5 of 10 patients (^18^FEC PET/CT).

It is assumed that ^68^Ga-PSMA-11 PET/CT reveals the highest contrast in lymph node metastases, followed by bone metastases, local relapses and soft tissue metastases [27]. ^68^Ga-PSMA-11 PET/CT allows the detection of bone and organ metastases – due to low background signal [19] – which may lead to systemic therapy, but if excluded may lead to curative therapy [41]. ^18^F-PSMA agents are an attractive alternative to ^68^Ga-PSMA compounds. ^18^F-PSMA can be produced in larger amounts per batch in PET radiopharmaceuticals with an on-site cyclotron. Additionally, the average lower positron range of ^18^F reduces blurring effects leading to a higher spatial resolution and the longer half-life of ^18^F (110 min.) in comparison to ^68^Ga (67 min.) optimizes the production and distribution of ^18^F [42].

## MATERIALS AND METHODS

### Patients

In this retrospective study we included 61 consecutive patients from the Central Military Hospital Koblenz and 4 consecutive patients from the Practice of Radiology and Nuclear Medicine Cologne Triangle with suspected prostate carcinoma due to an elevated PSA (≥4.0, depending on age). Patients with a history of specific cancer pretreatment, surgical intervention, or inconspicuous PSA plasma levels were excluded. In 25 patients we conducted a ^68^Ga-PSMA-11 PET/CT (7/2015 – 08/2016) and in another 40 patients an ^18^FEC PET/CT (2/2010 – 7/2015). In all patients, prostate cancer was verified histologically with transrectal ultrasound (TRUS)-guided biopsy, and the GS results of TRUS biopsy served as reference for the PET findings. Detailed information on patient characteristics is shown in Table 1. Biopsy specimens were histopathologically evaluated based on the Gleason System on ISUP criteria 2014 and stratified by categorization into low-grade (GS≤3+4 = 7a) and high-grade malignancies (GS≥4+3 = 7b), respectively [43].

Our study was in accordance with the Helsinki Declaration and with our national legislation (German Medicinal Products Act, AMG § 13 Abs. 2b), and all patients gave their written informed consent. The retrospective study was approved by the ethics committee (Landesärztekammer Rheinland-Pfalz).

### PET/CT imaging protocols

PET/CT acquisition was performed on a Biograph 64 TruePoint (True V HD) PET/CT scanner (Siemens, Erlangen, Germany) for the Koblenz patient group and on a Gemini GXL 16 (Philips, Eindhoven, NL) for the Cologne patient group.

^68^Ga-PSMA-11 PET/CT was performed about 60 min. (whole body) after intravenous injection of ^68^Ga-PSMA-11 (median 176 MBq, range 157-268 MBq). ^68^Ga-PSMA-11 was obtained from the Department of Nuclear Medicine of the University of Mainz and from Advanced Accelerator Applications Bonn.

^18^FEC PET/CT was performed about 60 min. (whole body) after intravenous injection of ^18^FEC (IASON, Linz, Austria; median 230 MBq, range 175-291 MBq).

A contrast-enhanced diagnostic CT scan (140 keV, 100-400 mAs, dose modulation) or a low-dose CT scan (120 keV, 55 mAs) was performed for attenuation correction at the time of the PET scan. Contrast media could not be used in the following cases: renal insufficiency, contrast media allergy, hyperthyreosis, and oral administration of the pharmaceutical Metformin. PET was acquired in 3D (matrix: 168x168/Koblenz;144x144/Cologne). Each bed position (axial field of view of 21.8 cm/Koblenz;19 cm/Cologne) was acquired for 3 min. Random, scatter and decay correction were applied to the emission data. An ordered-subsets expectation maximization (OSEM) algorithm was used for reconstruction (two iterations, fourteen subsets, Gaussian filtering, 4.2 mm/Koblenz;5.3 mm Cologne transaxial resolution, full-width at half-maximum). CT data were obtained for attenuation correction.

The uptake of ^68^Ga-PSMA-11 and ^18^FEC, i.e. the tracer concentration of the hypermetabolic cancer region detected in the image, was quantified in terms of SUV_max_.

SUV_max_ values above 2.5 were related to clear and reproducible visual detection of PET positivity and therefore a cut off of SUV_max_ 2.5 was used to discriminate PET positivity from PET negativity for both tracers [27].

### Statistical analysis

The PET/CT results were related to the GS obtained by histopathologic analysis of biopsies. Additionally, we analyzed correlations between PSA, SUV_max_, GS and detection of metastases.

The data analysis was performed using descriptive statistics (relative and absolute frequencies, arithmetic means, standard deviation).

Spearman rank correlation coefficients were calculated to specify the relationship between two variables. The SUV_max_ values were related to the GS obtained by histopathologic analysis of biopsies. Additionally, we analyzed correlations between PSA, SUV_max_, GS and detection of metastases.

By means of ROC analysis we tested the ability of the method to distinguish between high- and low-grade cancer (significant/not significant) by plotting the true positive cases (sensitivity) against the false positive cases (1-specificity) for various SUV_max_ cut off-levels. Area under the ROC curve (AUC) together with 95%-confidence interval (CI) and standard deviation (SD) were calculated to characterize the quality of the discrimination between the two groups.

All tests were carried out using the software BiAS; p-values of less than 0.05 were stated as significant.

## CONCLUSION

Our results show that ^68^Ga-PSMA-11 PET/CT and ^18^FEC PET/CT are both suitable for the detection of primary prostate cancer. An advantage for ^68^Ga-PSMA-11 PET/CT is its excellent imaging quality, its high specificity and a correlation of positive scans with GS which may allow a differentiation between low- and high-grade carcinoma. Our results support the view that ^68^Ga-PSMA-11 PET/CT promotes higher detection rates of significant malignancies requiring intervention as does the diagnostic procedure using ^18^FEC PET/CT.

Therefore ^68^Ga-PSMA-11 PET/CT is intended to increase diagnostic precision (avoiding false-negative results and understaging) to guide prostate biopsy and might help to reduce overtreatment of low-grade malignant disease as well as detect the foci of the highest Gleason pattern.

^68^Ga-PSMA-11 PET/CT is already clinically accepted in detecting metastases in patients with biochemical recurrence [31], but it may play an important role also in initial tumor staging similar to and in conjunction with mpMRI-supported biopsy [33, 42].

## Abbreviations

ADT: androgen deprivation therapy
^18^FEC: ^18^F-fluoroethylcholine
^68^Ga: ^68^Gallium
GS: Gleason Score
mpMRI: multiparametric magnetic resonance imaging
NPV: negative predictive value
PPV: positive predictive value
PSA: prostate-specific antigen
PSMA: prostate-specific membrane antigen
SUV_max_: maximum standardized uptake value
